# Association of dialysis facility-level hemoglobin measurement and erythropoiesis-stimulating agent dose adjustment frequencies with dialysis facility-level hemoglobin variation: a retrospective analysis

**DOI:** 10.1186/1471-2369-12-22

**Published:** 2011-05-20

**Authors:** Irfan Khan, Mahesh Krishnan, Anupam Kothawala, Akhtar Ashfaq

**Affiliations:** 1 Amgen Inc., One Amgen Center Drive, Thousand Oaks, CA, 91320-1799, USA; 2 DaVita Inc., 1551 Wewatta St., Denver, CO, 80202, USA

## Abstract

**Background:**

A key goal of anemia management in dialysis patients is to maintain patients' hemoglobin (Hb) levels consistently within a target range. Our aim in this study was to assess the association of facility-level practice patterns representing Hb measurement and erythropoiesis-stimulating agent (ESA) dose adjustment frequencies with facility-level Hb variation.

**Methods:**

This was a retrospective observational database analysis of patients in dialysis facilities affiliated with large dialysis organizations as of July 01, 2006, covering a follow-up period from July 01, 2006 to June 30, 2009. A total of 2,763 facilities representing 436,442 unique patients were included. The predictors evaluated were facility-level Hb measurement and ESA dose adjustment frequencies, and the outcome measured was facility-level Hb variation.

**Results:**

First to 99th percentile ranges for facility-level Hb measurement and ESA dose adjustment frequencies were approximately once per month to once per week and approximately once per 3 months to once per 3 weeks, respectively. Facility-level Hb measurement and ESA dose adjustment frequencies were inversely associated with Hb variation. Modeling results suggested that a more frequent Hb measurement (once per week rather than once per month) was associated with approximately 7% to 9% and 6% to 8% gains in the proportion of patients with Hb levels within a ±1 and ±2 g/dL range around the mean, respectively. Similarly, more frequent ESA dose adjustment (once per 2 weeks rather than once per 3 months) was associated with approximately 6% to 9% and 5% to 7% gains in the proportion of patients in these respective Hb ranges.

**Conclusions:**

Frequent Hb measurements and timely ESA dose adjustments in dialysis patients are associated with lower facility-level Hb variation and an increase in proportion of patients within ±1 and ±2 g/dL ranges around the facility-level Hb mean.

## Background

A central aim of anemia management in dialysis patients is to maintain patients' hemoglobin (Hb) levels consistently within a target range. Anemia management guidelines and protocols thus require appropriate adjustment of erythropoiesis-stimulating agent (ESA) doses in response to Hb deviations relative to the target range. There are varied opinions among clinicians, however, regarding practice patterns that help achieve this objective [1-7].

The current US Food and Drug Administration-approved ESA label recommends an Hb target range of 10 to 12 g/dL when treating dialysis patients [8,9], which represents a slightly broader range than the 11 to 12 g/dL Hb target currently recommended by the National Kidney Foundation [10]. Recent data from randomized clinical trials (RCTs) have suggested no benefit and raised concerns regarding deleterious effects observed with Hb targets of 13-15 g/dL in patients with chronic kidney disease who are treated with ESAs [11-14]. At the same time, however, there are adverse consequences associated with low Hb levels [15-21]. Thus, at the level of individual dialysis provider, Hb target ranges have been influenced by the need to balance these opposing risks with the benefits of achieving Hb levels within the 10 to 12 g/dL range.

Recently, the Centers for Medicare and Medicaid Services proposed a rule for the End-Stage Renal Disease Prospective Payment System that includes a payment penalty for dialysis facilities that maintain more than a specified proportion of patients in Hb <10 or >12 g/dL ranges [22]. It is therefore helpful for clinicians to understand the nature of anemia management protocols that could maximize the number of patients within the desired Hb target range. Our aim in this study was to use the historical cross-sectional and longitudinal variation in the facility-level practice patterns of Hb measurement and ESA dose adjustment frequencies to study their association with the facility-level Hb variation, which is a key summary measure of a facility's ability to maintain patients within a given Hb target range.

## Methods

A retrospective observational database was constructed from electronic patient-level data contributed by large dialysis organizations (LDOs) in the United States. An LDO was defined as a chain of >500 affiliated dialysis centers. All data were de-identified in accordance with the Health Insurance Portability and Accountability Act Privacy Rule for limited use datasets; thus no additional institutional review board approval was sought for this study. This database has been described in detail in a previous publication [23]. Data from July 2006 to June 2009 (36 months) were extracted from dialysis centers affiliated with an LDO as of July 01, 2006. A study time period of July 2006 to June 2009 was chosen because it coincided with a significant longitudinal variation in the facility-level Hb variation, Hb measurement frequency, and ESA dose adjustment frequency. This longitudinal variation was influenced in part by regulatory and reimbursement changes for ESAs during this time. Only hemodialysis (HD) patients with >30 days of data during this 36-month interval were selected. Included HD patients represented only the thrice-weekly in-center HD modality, while other variants of HD treatment (daily, home, nocturnal, etc.) were not included in the cohort. Although ESA administration was not considered an inclusion/exclusion criterion, the ESA type variable in the analysis dataset and available LDO protocol information indicated that all ESA administrations in the final study cohort were in the form of intravenous epoetin alfa.

The dialysis facility was regarded as the unit of analysis. For each dialysis facility, the following sets of variables were calculated for each month during the study. They were (1) Hb variation, defined as the SD of all Hb measurements across all patients; (2) the number of patient-days, defined as the summation across all patients of the following measure: date of last patient-level dialysis session - date of first patient-level dialysis session + (7/3), with 7/3 applied to adjust for the follow-up truncation at the last observation in a month, assuming the average sequence of dialysis sessions was 3 times per week (TIW) for HD patients; (3) Hb measurement frequency, defined as the number of Hb measurements across all patients divided by the number of patient-days/30; and (4) ESA dose adjustment frequency, defined as the number of ESA dose adjustments across all patients divided by the number of patient-days/30.

Only persistent dose changes were regarded as true clinical dose adjustments (i.e., those that appeared as a-a-a-b-b-b, as opposed to a-a-a-b-a-a, over a TIW sequence). Dose changes were considered to be persistent if they lasted for at least 3 TIW sessions for non-zero doses and at least 9 TIW sessions for zero doses. The longer time requirement for zero-dose adjustments helped reduce potential misclassification of intentional clinical adjustments to zero doses with hospitalization events. We validated the methodology with data from patients for whom hospitalization information was available (approximately 50% of the study cohort). The validation step indicated that this methodology would result in a very low proportion (approximately 0.3%) of dose adjustments that could be potentially misclassified. We believe that this level of error is unlikely to cause a meaningful impact on the results. The algorithm adopted for identifying dose changes considered the entire patient-level longitudinal data before further categorization by calendar months. The calendar month boundaries thus did not interfere with the definition of a dose change.

We summarized the aggregate variation in facility-level Hb variation, Hb measurement frequency, and ESA dose adjustment frequency by pooling these measurements across all facility-month combinations and calculating basic descriptive statistics. A continuous distribution curve for these measures was created by calculating the percentage of facility-months within specific intervals. By adopting a method similar to one in a previous publication [23], we produced a smooth approximation of the distribution curve to avoid unnecessary attention to density fluctuations in low-prevalence buckets. The longitudinal variation in these measures was summarized by calculating the mean (95% CI) values by each month across all facilities. The number of patient-days was introduced as a weight in these calculations.

We studied associations between facility-level Hb variation and Hb measurement frequency and between facility-level Hb variation and ESA dose adjustment frequency by using a mixed-modeling regression approach that partitioned cross-sectional and longitudinal information in the data [24-26]. This modeling framework was adopted to address the unavailability of facility-level characteristics, which was one of the main limitations of the analysis dataset. When conclusions regarding associations in this modeling framework are drawn from the longitudinal part of the model, the same facility will effectively act as its own control over time, and the primary source of bias will be limited to systematic longitudinal changes in aggregate facility-level characteristics. The technical details and underlying rationale behind this are discussed more fully in previous work using these models [24-26].

We considered 2 separate models for studying the associations between facility-level Hb variation and Hb measurement frequency and between facility-level Hb variation and ESA dose adjustment frequency due to the relatively high collinearity between Hb measurement and ESA dose adjustment frequencies. An additional reason for considering 2 separate models was that a model which simultaneously considered both these factors could introduce a conceptual possibility that the benefit from these factors in terms of their implications on Hb variation could be regarded as additive and can accrue independently. Though the benefit from Hb measurement frequency can be considered to accrue independently, the same cannot be said about the ESA dose adjustment frequency since any practical anemia management protocol would consider Hb measurement frequency to exceed the ESA dose adjustment frequency. The fixed- and random-effects part of these mixed models included an identical set of parameters representing an intercept term, a linear cross-sectional slope parameter, and a linear longitudinal slope parameter. The number of patient-days was introduced as a weight in the preceding models. The mixed models were implemented using Proc MIXED in SAS (SAS Institute Inc., Cary, NC, USA). All analyses were performed in SAS, version 9.1.3.

We consider the model relating facility-level Hb variation and Hb measurement frequency as not being influenced by an endogeneity problem due to omission of the number of Hb measurements from the model. This is because we defined Hb variation in terms of SD. The expectation of Hb variation is therefore independent of sampling more or less Hb observations, which would influence the precision of estimating the SD. We would highlight that we have not defined Hb variation as the precision of SD, but SD itself, which is a summary measure of the width of the facility-level Hb distribution curve.

Please see the Technical Appendix (Additional file 1) for further information regarding model formulation.

## Results

A total of 2,763 facilities were included in the analysis. They represented all LDO-affiliated facilities as of July 01, 2006, and accounted for approximately 60% of all dialysis facilities in the United States at that time. Data from 2,734 facilities (99% of the original cohort) were available for all 36 months of follow-up, with the remaining facilities (1%) lost to follow-up owing to closure. The total number of unique patients and facility-month combinations represented in the analysis dataset during the 36-month study period were 436,442 and 98,717, respectively.

**Figure 1:**
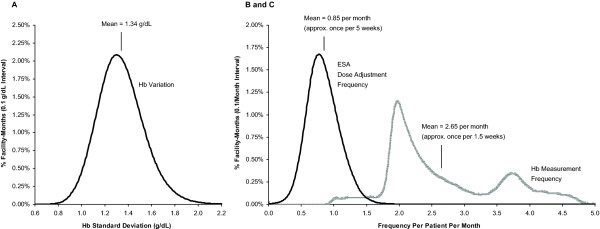
**Distribution of study variables across facility-months**. (A) Hb variation, (B) Hb measurement frequency, and (C) ESA dose adjustment frequency. ESA = erythropoiesis-stimulating agent; Hb = hemoglobin.

Figure 1A summarizes the distribution of facility-level Hb variation across facility-months. The mean facility-level Hb variation was 1.34 g/dL, with 1st and 99th percentiles ranging from 0.92 to 1.88 g/dL. Under the assumption of normality for Hb distribution curve at any point in time (corroborated in previous analyses considering a large cohort of patients [23,27]), the proportion of patients with Hb within ±1 and ±2 g/dL around the mean was higher for the 1st percentile behavior as compared with the 99th by approximately 32% and 26%, respectively (the percentage point difference represents p99 - p1, where p99 and p1 denote the proportion of patients in specific ranges for facility-months representing the 99th and 1st percentile behavior, respectively). The distribution of facility-level Hb variation was largely symmetrical about the mean with a slight positive skew (skewness factor, 0.49).

Figures 1B and 1C summarize the distributions of facility-level Hb measurement and ESA dose adjustment frequencies across facility-months. The mean facility-level Hb measurement frequency was 2.65 per month (approximately once per 1.5 weeks), with 1st and 99th percentiles ranging from 1.10 to 4.65 per month (approximately once per month to once per week). The distribution of facility-level Hb measurement frequency was bimodal with peaks at approximately 1.97 and 3.74 per month (approximately once per 2 weeks and once per 1 week). The distribution of facility-level Hb measurement frequency had a positive skew (skewness factor, 0.70), indicating a small proportion of facility-months with Hb measurement frequency significantly exceeding the mean. The mean of facility-level ESA dose adjustment frequency was 0.85 per month (approximately once per 5 weeks) with 1st and 99th percentiles ranging from 0.31 to 1.50 per month (approximately once per 3 months to once per 3 weeks). The distribution of facility-level ESA dose adjustment frequency was largely symmetrical about the mean with a slight positive skew (skewness factor, 0.39). There was a significant positive correlation between the distributions of facility-level Hb measurement and ESA dose adjustment frequencies with an estimated Pearson Correlation Coefficient of 30.7% (*P *< 0.001).

**Figure 2:**
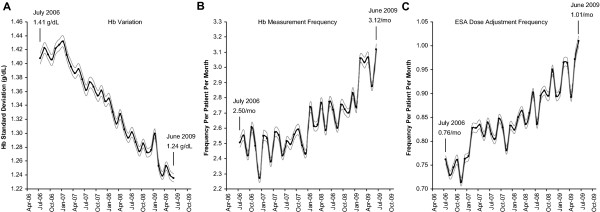
**Trend of the mean values of study variables by month across facilities**. (A) Hb variation, (B) Hb measurement frequency, and (C) ESA dose adjustment frequency. Bands represent 95% CI. ESA = erythropoiesis-stimulating agent; Hb = hemoglobin.

Figure 2 summarizes the longitudinal variation in the mean values of facility-level Hb variation, Hb measurement frequency, and ESA dose adjustment frequency. Mean facility-level Hb variation (Figure 2A) decreased from 1.41 g/dL (95% CI, 1.40-1.42 g/dL) in July 2006 to 1.24 g/dL (95% CI, 1.23-1.24 g/dL) in June 2009. Under the assumption of normality for Hb distribution curve at any time point, this indicated that on average a facility's ability to maintain patients within ±1 and ±2 g/dL around the mean during the study period increased by approximately 6% and 5%, respectively. Mean facility-level Hb measurement frequency (Figure 2B) increased from 2.50 per month (95% CI, 2.47-2.54 per month) in July 2006 to 3.12 per month (95% CI, 3.08-3.16 per month) in June 2009, while mean facility-level ESA dose adjustment frequency (Figure 2C) increased from 0.76 per month (95% CI, 0.75-0.77 per month) in July 2006 to 1.01 per month (95% CI, 1.00-1.02 per month) in June 2009.

Table 1 provides a summary of the estimates for fixed-effect parameters for the previously described models that evaluated the associations between facility-level Hb variation and Hb measurement frequency and between facility-level Hb variation and ESA dose adjustment frequency. For both models, the estimates for intercept, cross-sectional parameter, and longitudinal parameter were significant (*P *< 0.001). The estimates for cross-sectional and longitudinal parameters for both models were negative, which indicated a consistent negative association between facility-level Hb variation and Hb measurement frequency and between facility-level Hb variation and ESA dose adjustment frequency. The magnitude of associations as informed by the cross-sectional and longitudinal parameters was not markedly different for both of these models. Based on the mean values of Hb measurement and ESA dose adjustment frequencies, the difference between cross-sectional and longitudinal parameter estimates translated to a relatively low facility-level Hb variation difference of approximately 0.03 g/dL to 0.05 g/dL as measured by the standard deviation. Formal testing, however, indicated that the cross-sectional and longitudinal parameters were statistically different from one another with a *P*-value for the difference <0.001 for both models.

**Table 1:** Fixed-effect parameter estimates for mixed models

	Fixed-Effect Parameter Estimates* (95% CI)				
	
**Model**	** Intercept**			**Cross-Sectional Parameter**	**Longitudinal Parameter**
Facility-level Hb variation and Hb measurement frequency	1.558			−0.083	−0.066
	(1.543 to 1.573)			(−0.089 to −0.078)	(−0.070 to −0.062)

Facility-level Hb variation and ESA dose adjustment frequency	1.443			−0.133	−0.096
	(1.427 to 1.460)			(−0.154 to −0.112)	(−0.104 to −0.088)

These estimates assess the associations between facility-level Hb variation and Hb measurement frequency and between facility-level Hb variation and ESA dose adjustment frequency.

ESA = erythropoiesis-stimulating agent; Hb = hemoglobin.

*All *P *< 0.001.

**Figure 3:**
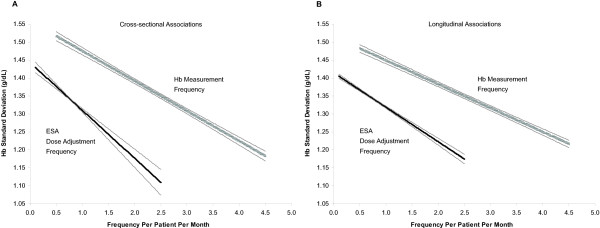
**Cross-sectional (A) and longitudinal (B) associations**. Associations shown are those between facility-level Hb variation and Hb measurement frequency and between facility-level Hb variation and ESA dose adjustment frequency, as informed by the fixed-effects part of the mixed model. ESA = erythropoiesis-stimulating agent; Hb = hemoglobin.

Figures 3A and 3B provide a graphical summary of the cross-sectional and longitudinal associations between facility-level Hb variation and Hb measurement frequency and between facility-level Hb variation and ESA dose adjustment frequency. These results indicate that under the assumption of normality for Hb distribution curve at any time point, an increase in Hb measurement frequency from once per month to once per week (from approximately 1st to 99th percentile behavior) was associated with an increase in the proportion of patients with Hb within the ±1 and ±2 g/dL around the mean by approximately 9 and 8 percentage points, respectively, according to the cross-sectional model, and by approximately 7 and 6 percentage points, respectively, according to the longitudinal model. Similarly, an increase in ESA dose adjustment frequency from once per 3 months to once per 3 weeks (from approximately 1st to 99th percentile behavior) was associated with an increase in the proportion of patients in these ranges by approximately 5 and 4 percentage points, respectively, according to the cross-sectional model, and by approximately 4 and 3 percentage points, respectively, according to the longitudinal model. When we modeled an increase in ESA dose adjustment frequency from once per 3 months to once per 2 weeks, the results were more consistent with the modeled increase in Hb measurement frequency, and the associated increases in the proportion of patients in these ranges were 9 and 7 percentage points, respectively, according to the cross-sectional model, and 6 and 5 percentage points, respectively, according to the longitudinal model.

## Discussion

We can suggest plausible mechanisms that may be responsible for driving the observed variation in facility-level anemia management practice patterns and their association with the facility-level Hb variation reported in this study. The relatively wide variations observed in facility-level Hb measurement and ESA dose adjustment frequencies suggest that physicians exercise a significant level of control over these practice patterns in their individual units. Thus, facilities with a higher rate of Hb monitoring may represent physicians who value an assessment of both point-in-time Hb measurement as well as the trend. Assessment of an Hb trend can be helpful; if the trajectory of Hb levels is suggestive of a systematic decline despite stable ESA dosing levels (potentially due to unanticipated changes in a patient's underlying clinical situation driven by concurrent clinical factors and a high level of comorbidity burden), protocols that encourage timely upward adjustment of ESA doses will be associated with better Hb control. Similarly, if the reverse situation occurs, ESA dosing protocols that encourage a timely reduction or temporary discontinuation of ESAs will result in significantly better control in managing unintended excursions towards the higher Hb ranges. If ESA doses are temporarily withheld to manage high Hb level excursions, the practice of frequent Hb monitoring also allows for a timely re-initiation of treatment before Hb levels decrease too much.

When the range of facility-level Hb variation from modeled scenarios was compared with the range represented in the 99th to 1st percentile behavior, it indicated that practice patterns considered in the current study represented only 20% to 30% of the potential for reducing the facility-level Hb variation. This is to be expected because there are a number of other unrelated factors and practice patterns that could influence the facility-level Hb variation. For example, one such practice pattern is the detailed nature of ESA dose adjustments as a function of specific Hb levels. Similarly, iron administration and iron indices can also influence response to erythropoietin: intravenous iron supplementation is independently associated with the risk of exceeding targeted Hb levels [28] and changes in intravenous iron administration have been associated with hemoglobin cycling [3]. These factors, though critically important in terms of how they could influence the facility-level Hb variation, are outside the scope of the current study.

The bimodal distribution of facility-level Hb measurement frequency reflects the fact that the most popular protocols concerning Hb measurement frequencies were either once per week or once per 2 weeks, with once per 2 weeks being the more prevalent protocol. The increase in mean facility-level Hb measurement frequency seen in Figure 2B reflects a systematic practice pattern shift to monitoring Hb levels more frequently, a behavior that was likely partially driven by changes in the regulatory and reimbursement environment concerning ESA treatment [23]. The alternating nature of mean facility-level Hb measurement frequency by month seen in Figure 2B could be the result of Hb measurement frequency being defined in this analysis at a monthly level, whereas the Hb measurement protocols that were actually being adopted by the dialysis facilities were defined in terms of time intervals counted by weeks. The increase in mean facility-level ESA dose adjustment frequency seen in Figure 2C reflected a systematic practice pattern shift towards adjusting ESA doses more frequently during the study period, from a frequency of approximately once per 6 weeks to once per 4 weeks, a behavior that was likely driven by the need for maintaining patients' Hb levels within a narrower target range. The same alternating pattern of mean facility-level ESA dose adjustment frequency by month was observed in Figure 2C with an underlying explanation similar to the trend for mean facility-level Hb measurement frequency in Figure 2B.

Our method of simultaneously modeling cross-sectional and longitudinal associations in the data is similar to that described elsewhere [24-26]. Details concerning the underlying methodology suggest that the same facility will effectively act as its own control over time in the part of the model informing longitudinal associations. Thus, the longitudinal part of the model helps offset the lack of facility-level covariates, which is one of the main limitations of our analysis data set. The primary source of bias in this case will be the systematic changes in aggregate facility-level characteristics, such as mean demographics and comorbidities, over time. However, these aggregate characteristics change over a much longer time horizon compared with the changes in the practice patterns considered in this study [29]. The cross-sectional and longitudinal parameter estimates were not markedly different from one another, with the difference translating to a facility-level Hb variation difference of approximately 0.03 to 0.05 g/dL as measured by the standard deviation, which could be considered as a value of low practical significance. This suggests that even a purely cross-sectional analysis for assessing these associations may be considered as being somewhat moderated from confounding influences. This type of moderation in confounding when an individual facility (as opposed to an individual patient) is regarded as the unit of analysis has been argued in previous studies that have used this approach in the context of dialysis patients [30,31] as well as other settings [32]. However, this perspective should be considered in light of results from formal statistical testing which indicated that the cross-sectional and longitudinal parameters were statistically different from one another, thus technically supporting the design of the current model simultaneously considering cross-sectional and longitudinal effects. We regard the facility-level framework adopted in this analysis, together with the modeling framework that partitioned the cross-sectional and longitudinal variation in the data, as methodologic advantages that could moderate the burden of confounding factors.

There are a number of important limitations of our study that warrant careful consideration. It is possible that the set of studied facility-level practice patterns did not exert a direct causal impact on Hb variation, but were surrogates for diligent anemia management that could have resulted in a simultaneously elevated likelihood of the implementation of the described practice patterns as well as a reduction in Hb variation owing to other unrelated factors. These unrelated factors, for example, could represent independent facility-level practice patterns implemented to address the need for managing Hb levels within a narrower target range over time. An important limitation of the analysis dataset was the unavailability of facility-level demographic characteristics or comorbidity information. However, we have provided a summary description of how the adopted modeling framework could have moderated this limitation. The modeling framework was able to effectively utilize the main strengths of the database, which included the availability of consistent longitudinal data at a patient-level and the ability to map patients to individual dialysis facilities. Other important limitations of the study include the fact that only HD patients within the LDO segment receiving intravenous ESA were represented, which limits the extrapolation of results to other patient and dialysis provider segments. Additionally, patients included in the analysis received only intravenous epoetin alfa and, consequently, these results should be considered valid only for this drug and route of administration. In addition, impact on facility-level Hb variation due to potential changes in patient-level iron markers and intravenous iron administration were not captured in the analysis due to unavailability of this data. We have implicitly assumed that the longitudinal changes in the Hb distribution curve at the facility-level during the study time period were primarily influenced by changes in practice patterns concerning Hb measurement and ESA dose adjustment administration frequencies.

## Conclusions

This study offers useful hypotheses regarding the nature of anemia management practice patterns in dialysis patients that could result in lower facility-level Hb variation and an increase in the proportion of patients within ±1 and ±2 g/dL ranges around the facility-level Hb mean. In particular, this study suggests that the practice patterns of frequent Hb measurements and timely adjustments of ESA doses could be helpful in attaining this objective.

## Competing interests

Drs. Khan and Ashfaq and Mr. Kothawala are employees of Amgen Inc. Dr. Krishnan is a former employee of Amgen Inc. and has also served on advisory boards and speaker bureaus for Amgen Inc.

## Authors' contributions

IK and AK analyzed the data in this study, and all authors interpreted the data. All authors contributed substantially to the writing of this manuscript and read and approved the final manuscript.

## Pre-publication history

The pre-publication history for this paper can be accessed here:

http://www.biomedcentral.com/1471-2369/12/22/prepub

## Supplementary Material

Additional file 1 **Technical Appendix**. This appendix contains additional description regarding the model formulation, covariance structure, parameter estimation, and confidence intervals. The appendix also contains residual diagnostics for assessing model adequacy and results from an unweighted analysis. Click here for file
