# Supplementary material for: Association of dialysis facility-level hemoglobin measurement and erythropoiesis-stimulating agent dose adjustment frequencies with dialysis facility-level hemoglobin variation: a retrospective analysis
Source: BMC Nephrol. 2011 May 20;12:22. doi: 10.1186/1471-2369-12-22 (PMC3123272; doi:10.1186/1471-2369-12-22)
Supplement: Additional file 1 — Technical Appendix. This appendix contains additional description regarding the model formulation, covariance structure, parameter estimation, and confidence intervals. The appendix also contains residual diagnostics for assessing model adequacy and results from an unweighted analysis. [file 1471-2369-12-22-S1.PDF]

Technical Appendix

Model Formulation

The model was formulated as:

$$Y_{ij} = [\beta_0 + \beta_C X_{i1} + \beta_L (X_{ij} - X_{i1})] + [b_{0i} + b_{Ci} X_{i1} + b_{Li} (X_{ij} - X_{i1})] + \varepsilon_{ij} \quad (1)$$

where subscripts i and j denote the facility and months, respectively, and subscript $j = 1$ denotes the baseline month of July 2006. The response variable Y denotes the facility-level Hb variation as measured by the standard deviation, and the predictor variable X denotes either the facility-level Hb measurement frequency or the ESA dose adjustment frequency for the two models under consideration. The set of terms under the first square parenthesis represent the fixed-effects part of the model, those under the second square parenthesis represent the random effects part of the model, and ε_{ij} represents the random error. Parameters β_0, β_C, and β_L denote the intercept, cross-sectional parameter, and the longitudinal parameter for the fixed effects, while parameters b_{0i}, b_{Ci}, and b_{Li} denote the same terms for the random effects. The cross-sectional model is obtained by setting $(X_{ij} - X_{i1})$ to zero, while the longitudinal model is obtained by setting X_{i1} to its mean value.

Covariance Structure

Equation 1 may be compactly written in the standard mixed-model notation as:

$$\boldsymbol{Y}_i = \boldsymbol{X}_i\boldsymbol{\beta} + \boldsymbol{X}_i\boldsymbol{b}_i + \boldsymbol{\varepsilon}_i \quad (2)$$

where the boldface symbols represent the vector analogues of quantities in equation 1. This model will automatically introduce the following so-called “Random Effects Covariance Structure” for $\boldsymbol{Y}_i$ (Fitzmaurice et al. 2004):

$$\text{Cov}(\boldsymbol{Y}_i) = \boldsymbol{X}_i \boldsymbol{G} \boldsymbol{X}_i' + \boldsymbol{R}_i \qquad (3)$$

where for the present case $\boldsymbol{G}$ represents a 3×3 symmetric matrix of 6 covariance parameters, and $\boldsymbol{R}_i$ is assumed to be a diagonal matrix with diagonal elements equal to σ^2 representing the residual error variance. Matrices $\boldsymbol{G}$ and $\boldsymbol{R}_i$ thus summarize the between-facility and within-facility sources of variation.

Parameter Estimation and Confidence Intervals

The confidence intervals in Figure 3 summarizing the cross-sectional and longitudinal associations represent the 95% confidence intervals for the estimated marginal expectation (or the estimated fixed-effects part) of $\boldsymbol{Y}_i$ which is equal to $\boldsymbol{X}_i\widehat{\boldsymbol{\beta}}$, where $\widehat{\boldsymbol{\beta}}$ represents the estimated value of $\boldsymbol{\beta}$. The 95% confidence intervals are calculated as $\boldsymbol{X}_i\widehat{\boldsymbol{\beta}} \pm Z_{0.975}\sqrt{\boldsymbol{X}_i\,\widehat{\text{Cov}}(\widehat{\boldsymbol{\beta}})\,\boldsymbol{X}_i'}$ where $\widehat{\text{Cov}}(\widehat{\boldsymbol{\beta}})$ represents the estimated covariance of $\widehat{\boldsymbol{\beta}}$. We utilized Proc MIXED in SAS (SAS Institute Inc., Cary, NC, USA) for model implementation which employs maximum likelihood (ML) and restricted maximum likelihood (REML) procedures for estimation. $Z_{0.975} = 1.96$ represents the z-value for 97.5% probability for a standard normal distribution. We have thus relied on the large sample properties of the sampling distributions of $\widehat{\boldsymbol{\beta}}$ and $\widehat{\text{Cov}}(\widehat{\boldsymbol{\beta}})$ for the purpose of the construction of the confidence intervals. Because of the large sample sizes in the underlying data, we consider this approximation to be an adequate one.

Residual Diagnostics for Model Adequacy

We assessed model adequacy by studying the scatter plot of residuals versus predicted values. Our aim in this exercise was to investigate any discernible trend or lack of randomness in the scatter plot, which could point to a lack of fit or other sources of inadequacy in the model. We studied the Cholesky transformed residuals and predicted values, which were obtained by specifying the VCIRY option in Proc MIXED in SAS. This transformation “de-correlates” the residuals and predicted values from a mixed-model, thus facilitating routine residual analysis for model adequacy.

Figure A1 is a scatter plot of transformed residuals versus transformed predicted values for the two models under consideration. In order to contain the amount of data in scatter plots, we limited the scatter plots to represent a random draw of 1,000 from 98,717 facility-months. We superimposed a LOESS curve on the scatter plot to aid the assessment of trend. The LOESS curve was based on the full 98,717 observations and was obtained via Proc LOESS in SAS. The scatter plots displayed no obvious systematic pattern, with a random scatter around a constant mean of zero. However, the LOESS curve exhibited a small deviation from being a horizontal straight line at zero level. The small trend in the LOESS curve suggested that inclusion of a quadratic term in the model could result in a marginally improved fit.

Unweighted Analysis

In order to test the sensitivity of results with respect to the number of patient-days (a marker of facility size) as a weight, we implemented a variation of the model without introducing the weight variable. The rationale for considering this alternate analysis was that if large facilities and small facilities potentially exhibited an intrinsically different relationship between Hb measurement or ESA dose adjustment frequencies and Hb variation, the use of weights could theoretically introduce bias. The results are summarized in Table A1 and indicate that they are comparable to results from the weighted analysis considered in the manuscript.

Reference

Fitzmaurice GM, Laird NM, Ware JH: Applied Longitudinal Analysis. New York, NY: John Wiley and Sons; 2004.

Table A1 – Fixed-effect parameter estimates for mixed models: Unweighted analysis

These estimates assess the associations between facility-level Hb variation and Hb measurement frequency and between facility-level Hb variation and ESA dose adjustment frequency.

	Fixed-Effect Parameter Estimates* (95% CI)		
**Model**	**Intercept**	**Cross-Sectional Parameter**	**Longitudinal Parameter**
Facility-level Hb variation and Hb measurement frequency	1.535 (1.518 to 1.551)	−0.077 (−0.083 to −0.070)	−0.062 (−0.067 to −0.058)
Facility-level Hb variation and ESA dose adjustment frequency	1.413 (1.396 to 1.431)	−0.102 (−0.124 to −0.080)	−0.083 (−0.091 to −0.075)

ESA=erythropoiesis-stimulating agent; Hb=hemoglobin.

*All *P*<0.001.

Figure A1 – Scatter plots and LOESS curves of transformed residuals versus transformed predicted values for (A) Model 1: Hb measurement frequency versus Hb variation, and (B) Model 2: ESA dose adjustment frequency versus Hb variation

The scatter plots (solid circles) are based on a random draw of 1,000 from 98,717 facility-months. The LOESS curve (solid line) is based on full 98,717 observations.

A

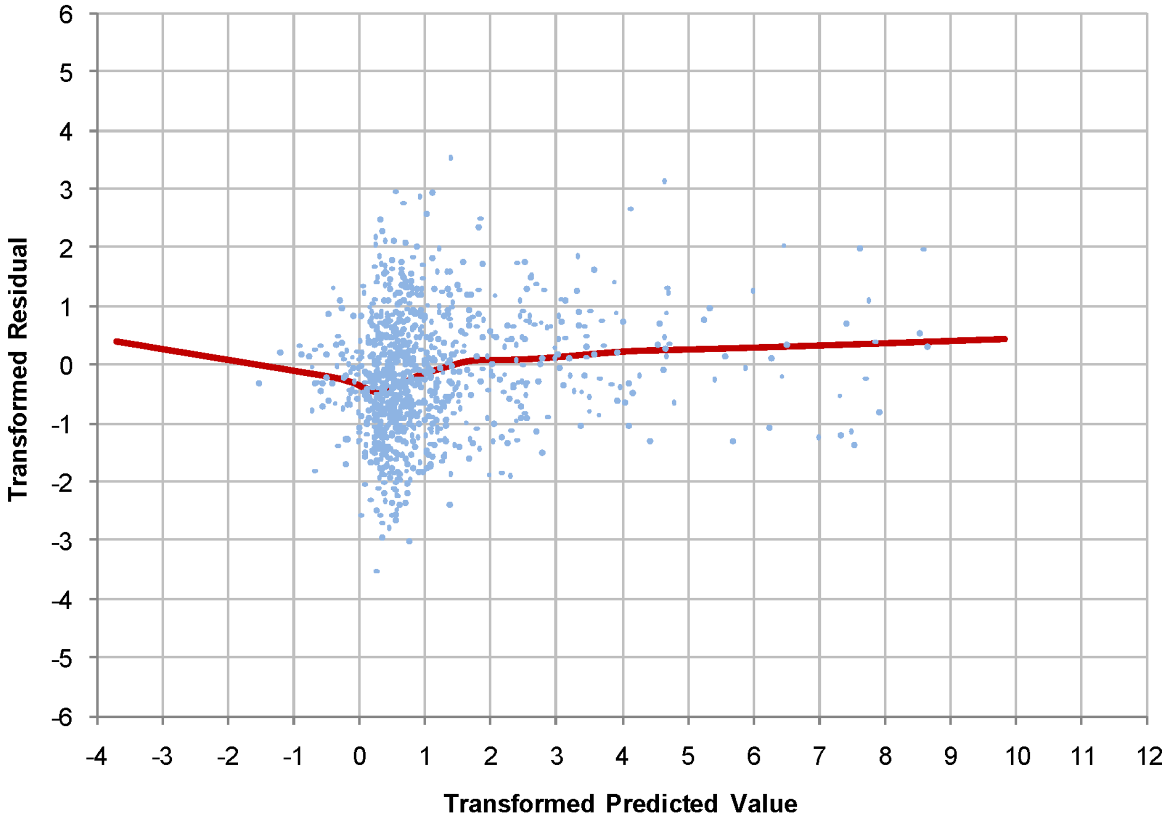

B

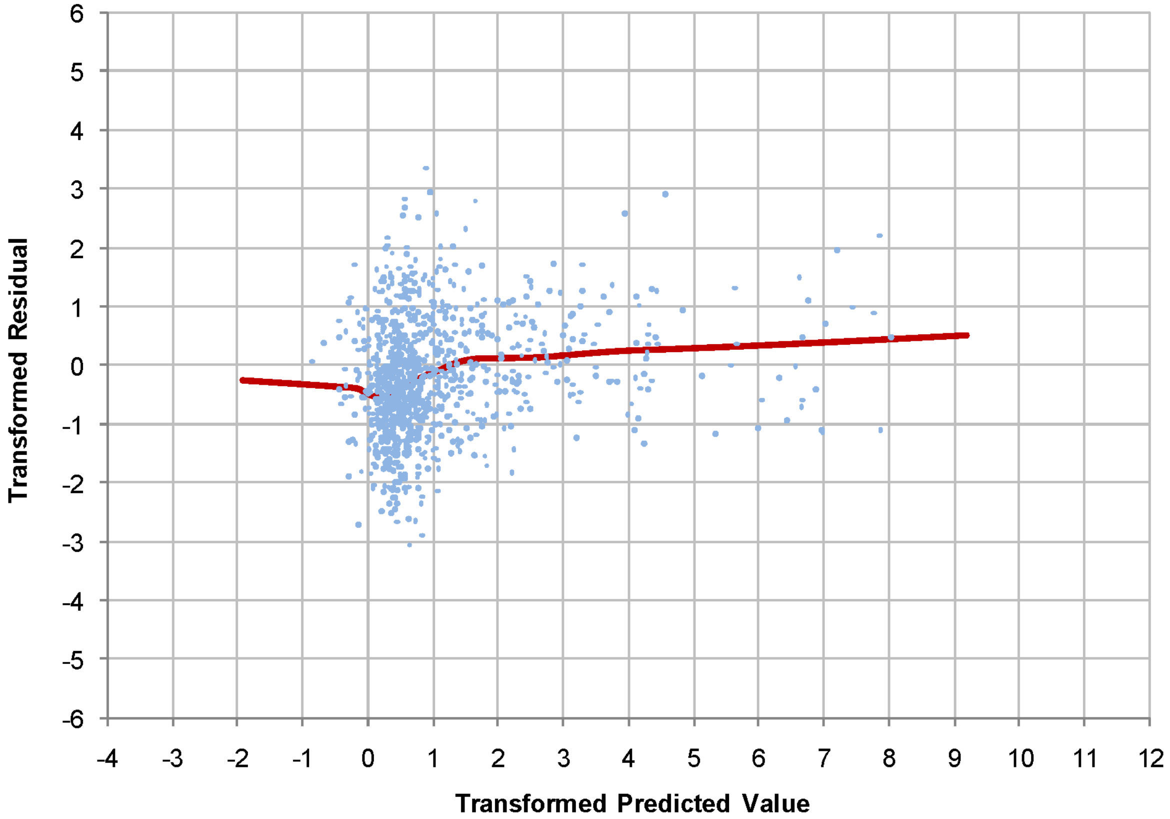
6
5
4
3
2
1
0
-1
-2
-3
-4
-5
-6
Transformed Residual
-4
-3
-2
-1
0
1
2
3
4
5
6
7
8
9
10
11
12
Transformed Predicted Value
